# Wetting and Spreading of AgCuTi on Selective Laser-Melted Ti-6Al-4V

**DOI:** 10.3390/ma14174804

**Published:** 2021-08-25

**Authors:** Lujing Hao, Jiankun Liu, Yulong Li

**Affiliations:** 1 Mechanical and Electrical Engineering School, Nanchang University, Nanchang 330031, China; 2 Key Lab for Robot and Welding Automation of Jiangxi Province, Mechanical and Electrical Engineering School, Nanchang University, Nanchang 330031, China; 18846440731@163.com (J.L.); liyulong@ncu.edu.cn (Y.L.)

**Keywords:** selective laser melting, Ti-6Al-4V (TC4), brazing, wetting and spreading, AgCuTi filler metal

## Abstract

Selective laser melting (SLM) can be used to manufacture complex parts, however, it is difficult to make large parts due to the size limitation of the SLM equipment. In application, smaller selective laser-melted (SLMed) Ti-6Al-4V (TC4) parts can be brazed or welded to form larger components. In the brazing, AgCuTi is often used to braze TC4. However, the wettability of AgCuTi on the SLMed TC4 should be evaluated before joining the SLMed TC4 parts. As a result, wetting and spreading tests and brazing experiments should be undertaken to successfully join the SLMed TC4 parts. In this study, a LINKAM TS 1500 high-temperature hot stage was used to test the brazability of the AgCuTi on the surface of SLMed TC4. Different temperatures and dwell times were used: (i) 850 °C 900 °C and 950 °C, holding for 120 s, were used to study the temperature effects; (ii) 20 s, 120 s and 200 s were used at 850 °C to study the dwell time effects. The R~t model was used to describe the wetting and spreading process. The results of this study can provide basic data for the joining of SLMed TC4 in industry.

## 1. Introduction

Three-dimensional (3D) printing (3DP), known as additive manufacturing (AM) is opposed to the “subtractive manufacturing” of raw materials used in traditional processing technology. It is based on digital model files, which allows the fabrication of structures in a layer-by-layer fashion. The 3DP technology generally integrates multiple disciplines, such as high-energy beam heat source, computer design, numerical simulation, numerical control, metallurgy and new materials etc., and realizes the mold-free, fast and near-net forming of complex 3D parts [1,2]. Three-dimensional printing technology has attracted the attention of many scholars at home and abroad, and has been widely used in aerospace, chemical metallurgy, construction, medical and other fields, due to its high processing accuracy, short manufacturing cycle, high material utilization ratio, and the ability to design and process parts with personalized requirements [3]. A variety of additive manufacturing technologies have been developed to date, among which SLM technology is particularly suitable for forming parts of titanium alloy, aluminum alloy and high temperature alloy due to its excellent printing efficiency and quality. SLM technology has also become one of the main ways of 3D printing for metal powder materials.

TC4 is one of the most popular materials in SLM technology because of its advantages of good corrosion resistance, high strength and low density, and has attractive application potential in aerospace industry and biomedical engineering [4]. SLMed TC4 is mainly composed of α’ martensite due to the ultrafast cooling rate in the SLM process, while conventional cast TC4 is composed of α and β phases [5]. The tensile, hardness and other mechanical properties of SLMed TC4 are close to or even better than those of traditional casting TC4 due to the presence of α’ martensite in a fine acicular slat [6]. However, SLM technology can only be applied in the manufacturing of small and medium-size complex components due to the limitation of forming process and the size of equipment, and it is difficult to meet the requirements of one-time forming process for large-size parts. 

With the development of SLM technology, the size of equipment is gradually increasing. However, it is still difficult to meet the market demand for large-size parts [7]. This problem can be solved well if several small and medium-sized SLMed components are joined together to form the final large-sized components. Therefore, it is necessary to study the joining technology of SLMed parts, which is of great significance to the development of SLM technology. 

Some scholars have recently conducted in-depth research on the method of joining small-size SLMed parts to larger parts. Prashanth et al. [8] used friction welding to join SLMed Al-12Si. The results show that the hardness of the material in the welding zone decreases, thus improving the overall ductility of the sample after friction welding. In another article, Prashanth et al. [9] successfully used solid-state welding processes to join SLMed TC4 components and improved the ductility, which demonstrates that solid-state welding processes can successfully join SLMed parts and improve the ductility. Nahmany et al. [10] studied the SLMed Al-Si-10Mg specimens formed by electron beam welding and first proved the feasibility of applying electron beam welding technology in SLM manufacturing. Tillmann [11] studied the joining of traditional manufactured and SLMed AISI 316L stainless steel using a vacuum brazing method. The fracture location of the joint of SLMed/conventional steel is at the flaws of pores and the joint strength is 317.4 MPa, which is 67.4% of that of conventional joint. The effects of oxides, inclusions and porosity in SLM process on the microstructure and mechanical properties were also evaluated. Results of the work of Tillmann show that brazing has the potential to be used to successfully join the SLMed parts with high accuracy. Xia et al. [7] used BNi2 filler metal to vacuum braze SLMed Inconel 718. The results show that the filler metal has good wetting and spreading on the SLMed Inconel 718, and SLMed nickel has good brazability. Yu et al. [12] successfully joined SLMed/rolled TC4 and SLMed/SLMed TC4 specimens by using the laser welding method, and they studied the microstructure, microhardness, tensile properties, fatigue life and fatigue crack growth rate. This proves that SLMed TC4 has good laser weldability after stress removal. 

Brazing is one of the most commonly used assembly methods for different materials among all the joining methods because of its convenient process and high accuracy, also the ability of completing multiple joining parts in a one-time heating process [13]. In the application of the brazing process, the wettability of the molten filler metal on the substrate is the decisive factor for the quality of the brazed joint. The wetting spreading of the brazing filler metal on the base metal during brazing has a great influence on the formation of the brazed joint [14,15]. Thus, it is necessary to quantitatively model and predict the wetting phenomena of the molten liquids of brazing filler metal on the solid substrates, in order to control the brazing process and improve the joint quality. However, it is difficult to establish a comprehensive theoretical model to describe the wetting behavior of liquids on solid substrates, since there are some chemical and physical reactions in the wetting spreading process, such as element diffusion, substrate dissolution and interfacial reaction between solids and liquids [16,17]. 

Many researchers have focused on the wetting behavior of liquid metal on the TC4 substrate in traditional casting or rolling state. In our previous work, Yu et al. [18] studied the influence of temperature on the wetting behavior of AgCu filler metal on conventional rolled TC4 in argon atmosphere and found that the increase of temperature significantly improved the wetting behavior of the filler metal. Liu et al. [19] studied the influence of high temperature on BNi2/SLMed TC4 system by using BNi2 nickel-based filler metal with a higher melting point than the α/β transformation temperature of TC4. However, in this high-temperature wetting system, the temperature had limited effects on the wetting process, and the wetting at high temperatures had significant corrosion effects on the substrate due to the dissolution of TC4 in the nickel. On the other hand, for the wetting mechanism itself, Li et al. [20,21] studied the influence of surface roughness on the wetting and brazing of the AgCu/TiAl system. They found that the wetting kinetics was mainly controlled by the dominant chemical reaction in the triple line region. Liu et al. [22] found the appearance of the precursor film in the front of the triple line, and the mechanism was characterized by modeling. Among the above listed literatures, AgCuTi is the most commonly used active filler, which is widely applied in the brazing of ceramics and metals. The active element Ti in the filler can react with oxides of ceramics thus having good wettability to ceramic substrates. The brazing of TC4 and ceramic has been widely studied. However, the microstructure of traditional rolled TC4 is different from that of SLMed TC4, which leads to the difference of their properties. Therefore, research on the wettability of the selected active brazing filler metal (AgCuTi) on TC4 and SLMed TC4 can provide effective guidance information for the readers. 

In this study, we design a series of experiments with different wetting temperatures, and holding times, on the basis of the previous research. The aim is to study the influences of the temperature and dwell time on the wetting process and interfacial structure of the AgCuTi/SLMed TC4 system, which may provide a good reference for accurately control the joining of the SLMed parts. The results in this study may have potential applications in automotive, aerospace, national defense, medical and other fields, in terms of large-size SLMed parts assembly.

## 2. Methods

**Figure 1:**
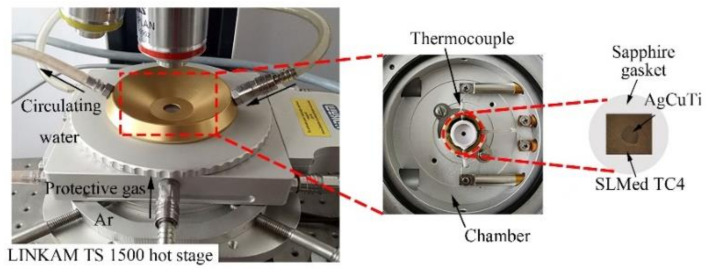
Schematic diagram of the high-temperature hot stage.

AgCuTi (Ag-Cu-4.5Ti, wt.%) with the melting point of 799.5 °C was used as the filler metal, SLMed TC4 was used as the base metal. 600 #, 1200 # and 1500 # sandpaper were used to randomly grind the SLMed TC4 to remove the oxides on the surface. Then, random scratches in different directions were obtained on the surface. Before experiments, the substrates and filler metals were ultrasonically cleaned in acetone, rinsed with distilled water, and dried by hot air blast. Figure 1 is the schematic diagram of LINKAM TS 1500(Linkam Scientific Instruments Ltd., Tadworth, UK) high temperature hot stage used in the wetting experiments. The cleaned AgCuTi filler metal was placed on the center of the SLMed TC4 surface, then the samples were placed on the heating stage of the heating chamber. The upper cover was tightened, the temperature program was set, and the top optical microscope (Olympus BX51M, Tokyo, Japan) was used for real-time in situ observation and real-time recording of the wetting process. The parameters of selective laser remelting in the manufacturing process of SLMed TC4 are shown in Table 1. Details of SLMed TC4 are shown in Table 2. All wetting tests were carried out in the protective atmosphere of ultrahigh-purity argon (99.999%). In order to investigate the effects of temperature on the wetting behavior of AgCuTi on SLMed TC4, three peak temperatures were set at 850 °C, 900 °C and 950 °C, respectively, holding for 120 s; in order to study the effect of holding time on the wettability of the system, peak temperature of 850 °C was selected, holding for 20 s, 120 s and 240 s, respectively. 

The heating chamber was continuously filled with argon for 10 min before heating to ensure that the air in the heating chamber was discharged to prevent oxidation during the heating. During the heating process, the digital camera system recorded the wetting process with 22 frames per second. The heating rate of all wetting tests was set at 120 °C/min and then cooled down at 100 °C/min. A holding time of 1 minute was set at 700 °C in order to reduce the temperature lag effect, which made the filler metal and substrate heat evenly. At least five experiments were repeated for each different temperature to confirm the results. The video recording began when the temperature reached the neighbouring of the melting point. Virtual dub 1.10.5 software(originally used by Microsoft Windows) was used to extract individual frames from the collected video, and then each frame corresponded with temperature and time one by one. Image-Pro Plus 6.0(Media Cybernetics, Inc.) software was used to measure the spreading instantaneous photos of the molten droplets, and the relationship curve between the equivalent radius of droplets and time was obtained. The wetting spreading performance on the substrate was then analyzed. 

The calculation of equivalent radius during wetting and spreading is as follows: (i) recording the number of pixels of the scale; (ii) recording the number of pixels of the spread area; (iii) calculating the equivalent radius of the instantaneous trajectory of the solid droplet using the following equation:

R = A/B × (C/Π) ^1/2^ (1)

where R is the equivalent radius, A is the real length of the scale, B is the number of pixels in the scale, and C is the number of pixels of the spread area. 

## 3. Results and Discussion

### 3.1. Wetting Spreading Process and the Kinetics Analysis

#### 3.1.1. Influence of Temperature on the Wetting Process

**Figure 2:**
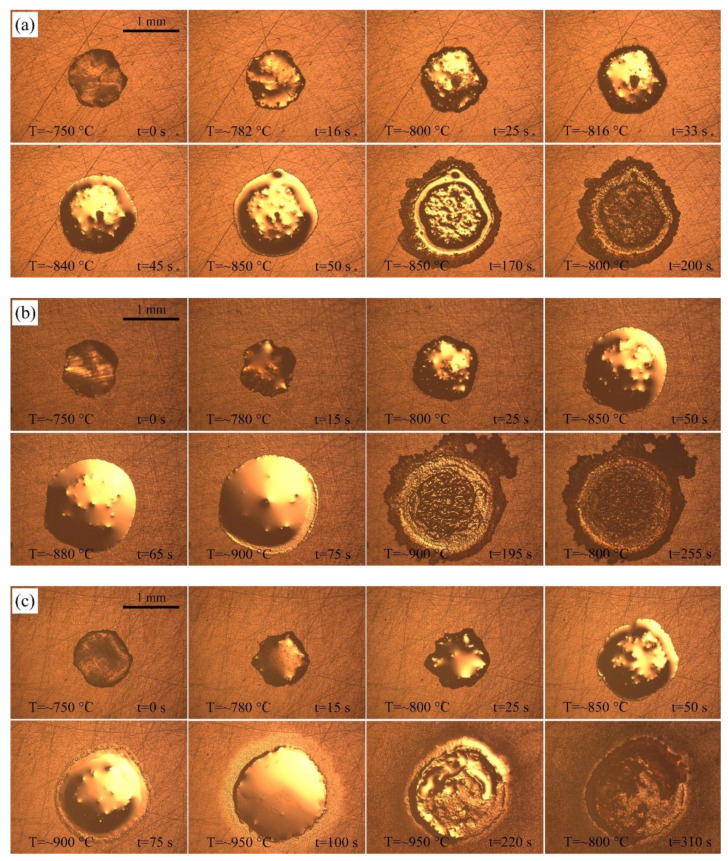
Typical instantaneous photos of AgCuTi wetting and spreading process on SLMed TC4 substrate for 120 s under different heating temperatures: (**a**) 850 °C; (**b**) 900 °C; (**c**) 950 °C.

Figure 2 shows the typical instantaneous photos of AgCuTi fillers on SLMed TC4 substrates during wetting and spreading. When the temperature is close to the melting point of AgCuTi, the size of the filler decreases slightly. Then, the temperature further increases, the filler metal can be observed to melt and spread around until the maximum spreading limit is reached. Finally, the filler metal solidifies on the surface of SLMed TC4 to form a residual filler after a series of changes such as melting, diffusion and reaction during the heating and cooling processes. 

Comparing the figures shown in Figure 2, it can be clearly observed from the results of 850 °C and 900 °C that: (i) the molten filler metal undergoes a process of slightly decreasing, and then rapidly spreading out; (ii) the central region of the molten liquid metal presents metallic luster, and there are some irregular protrusions in the central region; (iii) at the late stage of the holding process, a circle of black annular band arises. However, the results acquired at 950 °C are slightly different. The filler metal shrinks slightly at the beginning and then spreads out rapidly. Irregular protrusions appear in the central of the molten filler metal, then gradually disappear and the surface of the liquid filler becomes smooth. Moreover, after reaching the peak temperature, a shining precursor film quickly spreads out on the outer ring of the molten filler, which has also been found in Liu [22] and Zhao [23]. 

**Figure 3:**
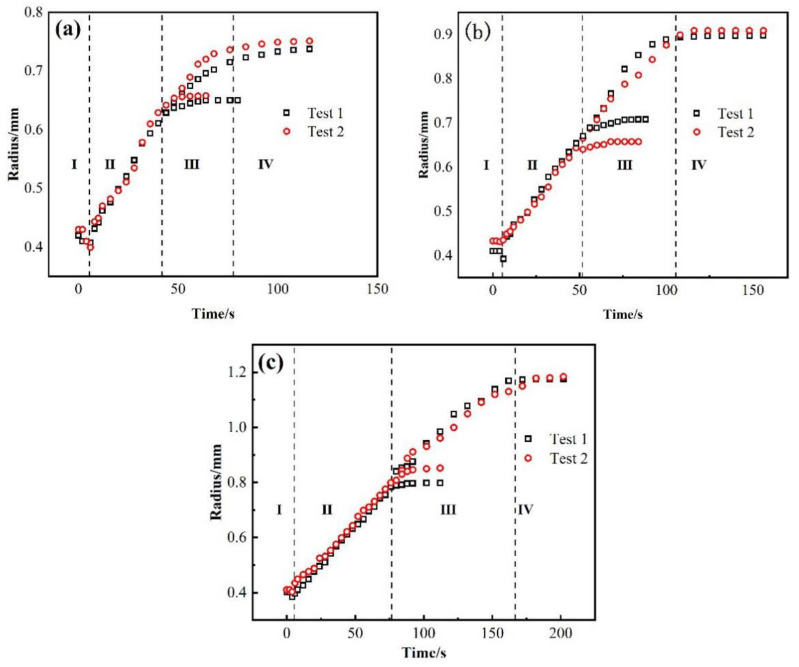
Relationship between the equivalent spreading radius and time of AgCuTi/SLMed TC4 wetting system: (**a**) 850 °C/120 s; (**b**) 900 °C/120 s; (**c**) 950 °C/120 s.

Figure 3 shows the relationship between the equivalent spreading radius and time of the AgCuTi/SLMed TC4 wetting system acquired at 850 °C, 900 °C and 950 °C, respectively. The results show that the time of the wetting process increases with the increase of temperature, and the wetting area increases accordingly. The precursor film phenomenon is more obvious, the spreading speed is faster, and the spreading range is wider at higher temperature than that of the lower temperature.

The wetting processes of AgCuTi of 850 °C and 900 °C are similar, and can be divided into four stages: (I) Initial spreading stage. As the temperature increases to the melting point of AgCuTi, the filler begins to change from solid state to liquid state with slight shrinkage. The reason may be the slight oxidation of SLMed TC4 substrate and AgCuTi surface in the process of sample preparation, which leads to the fact that the filler is in direct contact with the oxide layer on the substrate surface at the beginning [24]. At this time, the cohesion of AgCuTi is greater than the adhesion between the solid and the liquid due to the free energy of the solid/liquid interfacial tension, which induces the filler to shrink and form a larger contact angle. However, it can be seen from Figure 3 that this process only lasts 1–2 s, and then quickly enters the next stage. (II) Rapid spreading stage. With the increase of temperature, the diffusion and interaction between elements promote the decomposition of the surface oxide film, resulting in the decrease of the solid-liquid interfacial tension, which makes the molten liquid AgCuTi directly contact with the substrate, and continues to promote the formation of chemical reaction between Cu and Ti and other elements. At this stage, with the increase of temperature, the chemical reaction rate is faster, the diffusion of filler metal on the substrate gradually accelerates, and the filler metal spreads out rapidly. The main reason is that the chemical reaction takes place on the interface to form a new phase, which leads to the decrease of the solid-liquid interfacial tension and promotes the wetting spreading process. (III) The precursor film spreading stage. When the temperature reaches the set peak temperature, the central region of the molten filler hardly changes over time. Only the precursor film begins to spread out during the holding period. It can be seen from Figure 2a,b that the spreading area of the precursor film at the peak temperature of 900 °C is larger than that at 850 °C, and the shape of the precursor film is affected by the surface morphology of the substrate. This result can be explained by the fact that the main component of the precursor film is a thin layer of liquid Ag, and the roughness of the substrate hinders its spreading. (IV) The stage approaching balance. With the continuous wetting, the reaction between the filler and the substrate gradually declines, and the spreading of the precursor film also slowly stops, showing a trend of stable equilibrium. Finally, the molten filler reaches the maximum spreading area, and the whole wetting spreading process ends. However, the wetting process of AgCuTi on SLMed TC4 substrate is slightly different at 950 °C compared with the results of 850 °C and 900 °C, which can be divided into four stages. (I) Initial spreading stage. (II) Rapid spreading stage. (III) Stage of the bulk liquid slow spreading and precursor film fast spreading. In this stage, the two processes, bulk liquid slow spreading and precursor film fast spreading take place at the same time. The spreading speed of the precursor film is very fast since the temperature continues to rise. The area of the precursor film is much larger in the spreading result of 950 °C than that of 850 °C and 900 °C. 

**Figure 4:**
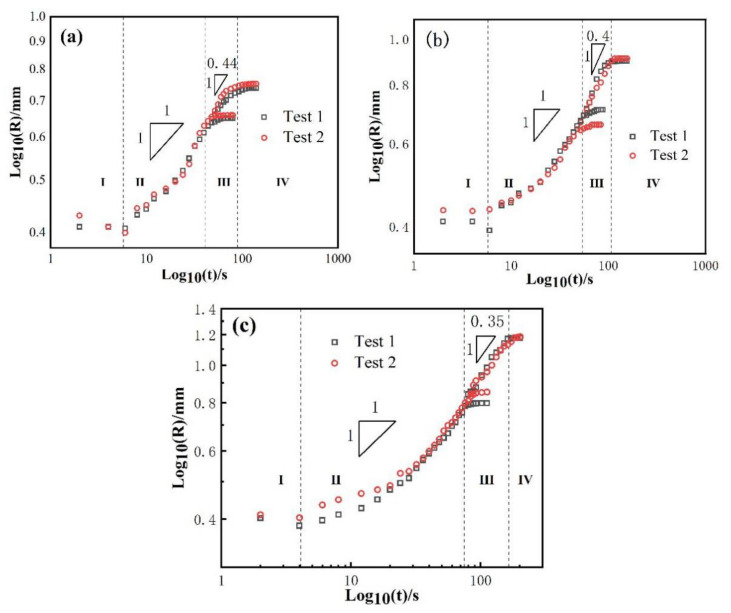
Logarithmic relationship between equivalent spreading radius and time of AgCuTi/SLMed TC4 wetting system: (**a**) 850 °C/120 s; (**b**) 900 °C/120 s; (**c**) 950 °C/120 s.

As mentioned above, the wetting spreading kinetics is mainly described by Washburn’s empirical power law model R^n^ ~ t. Tillmann et al. [25] think that when the diffusion rate of elements is higher than the reaction rate at the triple line, the wetting kinetics is limited by the reaction, which belongs to reaction limited wetting (where n = 1), and the wetting spreading time is about 10^1^~10^4^ s. When the diffusion rate of elements is less than the reaction rate, Landry [26] and Mortensen [27] believe diffusion to be the main limiting factor, which belongs to diffusion limited wetting (where n = 4), and the wetting spreading time may be only a few milliseconds. In order to better analyze the wetting mechanism of the AgCuTi/SLMed TC4 system, the equivalent wetting spreading radius of AgCuTi/SLMed TC4 system under three temperatures were measured, and the logarithmic relationship between the equivalent wetting spreading radius and time was obtained, as shown in Figure 4.

Under the three wetting temperatures, the main spreading of liquid on SLMed TC4 substrate follows the R^n^ ~ t relation of n = 1, which means that the spreading of molten liquid filler is mainly controlled by a chemical reaction. Landry [28] et al. show in the study of pure aluminum/glassy carbon system that the spreading of molten liquid filler metal is controlled by a chemical reaction, and similar wetting spreading phenomenon is observed at 827 °C and 917 °C. Similarly, in the Sn-35Bi-1Ag/Cu wetting system, Li [29] and others find that the n values of the corresponding spreading stages are similar at 210 °C and 260 °C, which is consistent with the experimental results of this study. In the spreading process of the precursor film, temperature has a great impact on it. It can be seen that the n value of the precursor film is significantly smaller than that of the bulk liquid (main liquid phase of the molten filler), which indicates that the spreading of the precursor film is much faster than that of the bulk liquid. The n value of the precursor film is about 0.44 at 850 °C, then it decreases to 0.4 at 900 °C and 0.35 at 950 °C. Precursor film can be found in many wetting systems, such as Sn-based alloy/Sialon ceramics [30], Zr-based alloy/ZrC [31], Al6061 or Al4043/steel [32]. In the AgCuTi/SLMed TC4 system, the formation of precursor film is considered to be the movement of Ag atoms in liquid. Firstly, the liquid Ag atoms are separated on the surface of the liquids and adsorbed on the edge of the triple line. Then, Ag atoms accumulate on the triple line with time. When Ag atoms accumulate to a certain amount, they suddenly overflow to the surface of the substrate and form a precursor film [22].

#### 3.1.2. Effect of Dwell Time on the Wetting Process 

In addition to the wetting temperature, the dwell time also affects the wetting process, and then affects the subsequent brazing/joining process. According to the results of wetting experiment in Section 3.1.1, the wetting and spreading is good at the peak temperature of 850 °C, 900 °C and 950 °C for 2 min. In order to accurately control the flow of molten filler metal, it is necessary to study the holding time effects. In some cases, in order to accurately control the flow of the molten filler metal, it is necessary to set a relatively low temperature to prevent an excessive wetting. Thus, the temperature of 850 °C is selected at the peak temperature to study the effects of the dwell time on the wetting processes.

**Figure 5:**
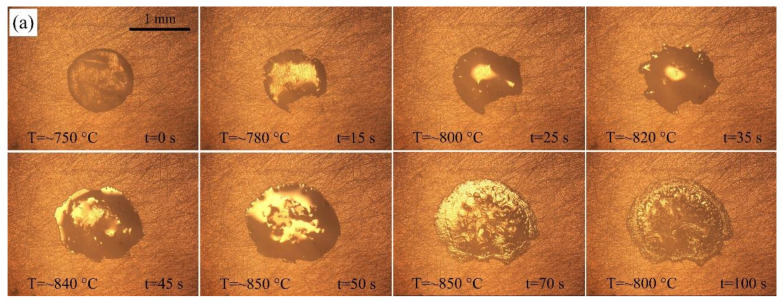

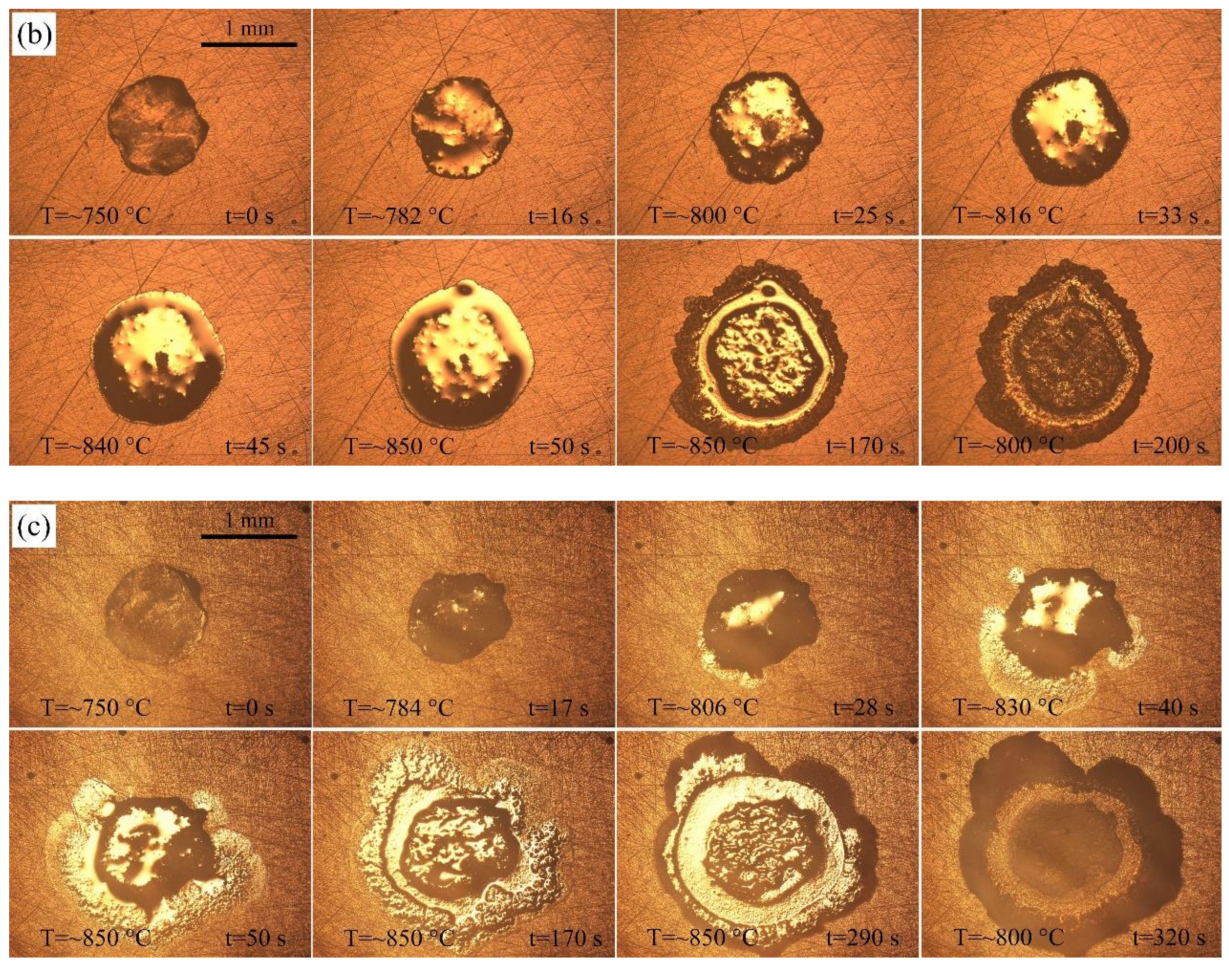
The representative instantaneous photos of wetting and spreading process of AgCuTi on SLMed TC4 substrate conducted at 850 °C with different dwell time: (**a**) 20 s; (**b**) 120 s; (**c**) 240 s.

**Figure 6:**
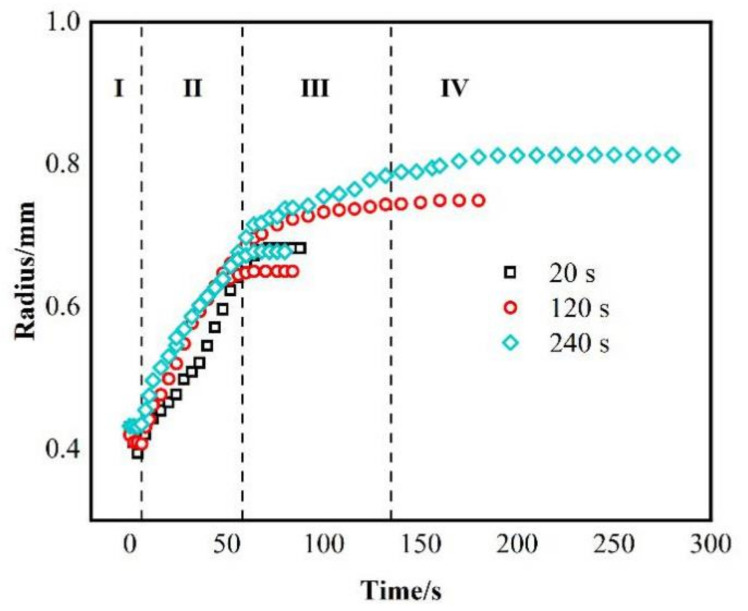
Relationship between the equivalent spreading radius and time of AgCuTi/SLMed TC4 wetting system under different holding time at 850 °C.

Three different holding times, 20 s, 120 s and 240 s, are set at the peak temperature of 850 °C to study the effect of holding time on the wetting spreading behavior of AgCuTi on the SLMed TC4 surface at a certain temperature. Figure 5 shows the typical instantaneous photos of AgCuTi during wetting and spreading on SLMed TC4 substrate at 850 °C for three different holding times. It can be seen from the figures that when the temperature is close to the melting point of AgCuTi, the size of the filler metal decreases slightly. When the temperature increases further, the molten filler melts and spreads out, and continues to spread with the increase of temperature and holding time until the maximum spreading limit is reached. By observing and comparing the figures in Figure 5, it can be clearly observed that when the holding time is 20 s, the wetting spreading area of the filler is relatively small. When the holding time is 120 s, the wetting spreading area is larger than that of the 20 s. When the holding time is extended to 240 s, it is obvious that the molten filler is even larger. Figure 6 shows the relationship between the wetting equivalent spreading radius and time of AgCuTi on SLMed TC4 at 850 °C for different holding times. The results show that the wetting process is similar to the previous results. With the increase of temperature, the filler firstly shrinks slightly and then spreads out until it reaches the peak temperature, and then continues to spread during the holding time. The final wetting area increases with the increase of holding time. It is worth noting that there is almost no precursor film phenomenon when the holding time is 20 s, but the precursor film phenomenon is more obvious when the holding time is 120 s and 240 s, and the spreading area of the precursor film is larger of 240 s than that of 120 s. However, all the precursor film in the spreading of the molten filler at 850 °C is obviously smaller than that of the 900 °C and 950 °C in the previous sections, which shows that the prolongation of holding time can slightly promote the spreading of molten filler, but the promoting effect is far less than that of the increase of temperature in this study. 

**Figure 7:**
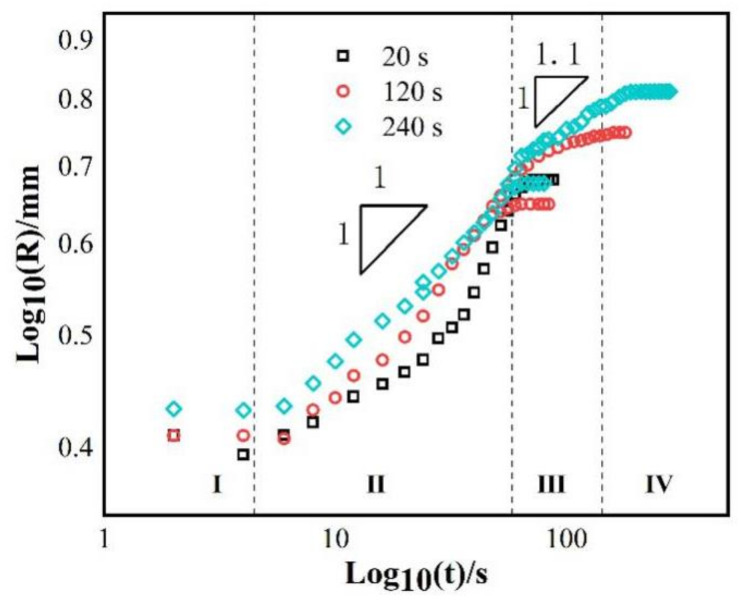
Logarithmic relationship between the equivalent spreading radius and time of AgCuTi/SLMed TC4 wetting system at different holding times conducted at 850 °C.

In order to further analyze the wetting mechanism of the AgCuTi/SLMed TC4 system, the logarithmic relationship between the equivalent spreading radius and time under three holding time at 850 °C is drawn, as shown in Figure 7. In the rapid spreading stage, the spreading of liquid filler on SLMed TC4 substrate follows the R^n^ ~ t relation of n = 1, which is similar to that of the results in Figure 4, the wetting and spreading at 850 °C, 900 °C and 950 °C for 120 s; but for the spreading of the precursor film, n = 1.1, which is different from the n value in the spreading stage of precursor film in the previous section. The reason for this difference is that the spreading of the precursor film occurs during the temperature holding process, at which time the temperature does not increase anymore, while in the previous section, the spreading of the precursor film is in the temperature rising stage, during which the temperature continuously rises to 900 °C or even 950 °C, which greatly promotes the spreading of the precursor film. 

### 3.2. Microstructure Analysis of Wetting Interface

**Figure 8:**
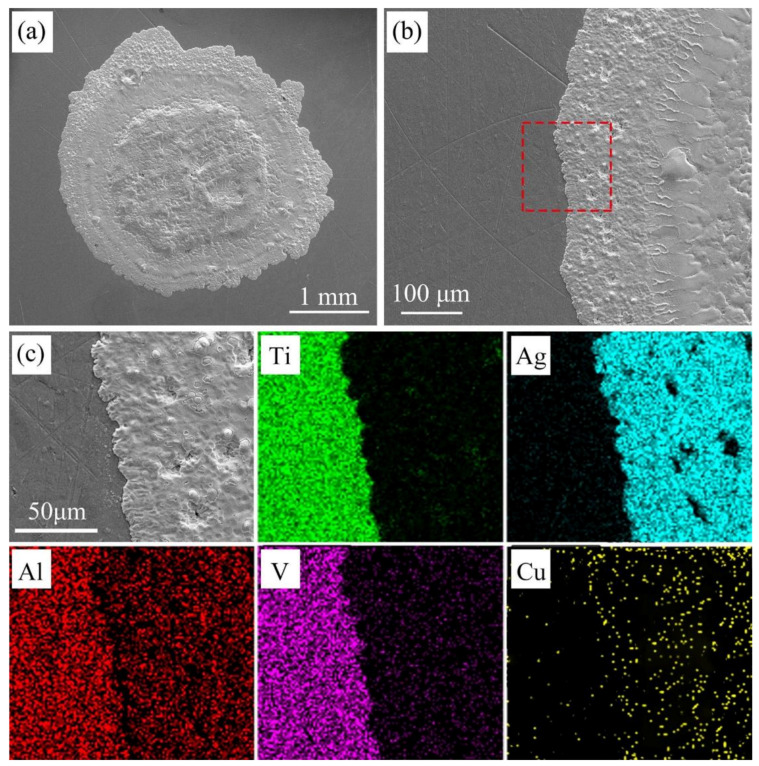
Scanning electron microscope (SEM) images and the associated elemental mappings of the samples (**a**) typical top view of the wetted samples; (**b**) enlargement of the triple line area; (**c**) element analysis results by energy-dispersive X-ray spectroscopy (EDS) mapping.

A typical top view of the wetted sample is showed in Figure 8a. It can be observed that after wetting, the filler metal spreads outwards almost in a circular manner, in which the slightly protruding part in the middle of the spreading filler metal is similar to the shape of the original filler metal foil, while irregular shape changes occur in the outermost ring. As the wetting energy of the filler metal is not sufficient to drive the molten filler going across the random protrusions of the front edge when it reaches the far-end edge, the filler metal can only spread along the grooves, thus forming an irregular shape. Figure 8b is the enlarged edge of the triple line, and a surface scanning analysis of the area in the red box is conducted. The element distribution is shown in Figure 8c. From the element mapping scan results, it can be seen that most of the elements near the triple line of the resolidified filler are Ag elements. It can be determined that the circle of black ring bands in Figure 2a are composed of Ag elements, which are also the main components of the precursor films. 

**Figure 9:**
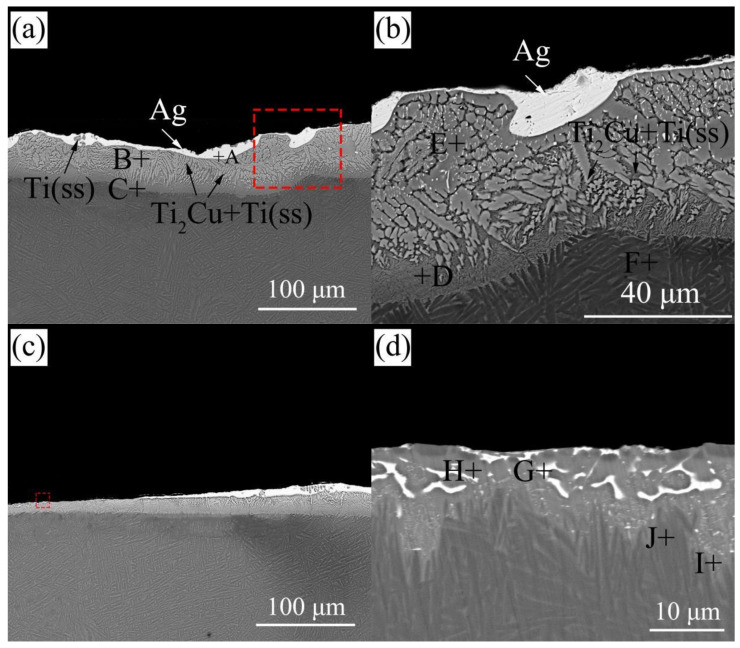
Microstructures of the wetted central area and triple line front area (**a**) central area of the cross-section of the wetted sample; (**b**) magnification of the red area in (**a**); (**c**) edge domain of the cross-section of the wetted sample; (**d**) magnification of the red area in (**c**).

According to the experimental results in Section 3.1.1, the wetting spread was good and typical under the condition of 900 °C/holding for 120 s. Therefore, we selected the samples obtained under the condition of 900 °C/120 s for analysis. The wetted specimens at 900 °C/120 s were cut through the central of the resolidified filler metal and the cross-section was obtained as shown in Figure 9. Figure 9a,c are the typical microstructures of wetted central area and triple line front area, respectively. Figure 9b,d show enlarged images of the areas marked in the red boxes in Figure 9a,c, respectively, to facilitate a clearer observation and analysis of the structure morphology. It can be seen that almost all of the top of the resolidified filler metals are coated with a white/bright Ag phase, extending to the edge of the triple line. 

**Figure 10:**
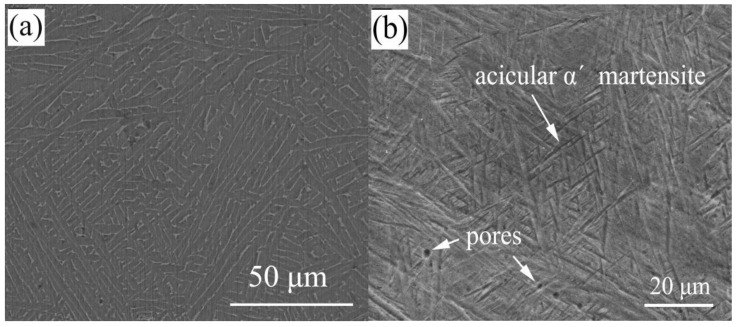
Microstructure of the SLMed TC4: (**a**) after wetting; (**b**) before wetting.

Composition analysis of different areas of the interface is performed under an electron microscope assisted by EDS. Table 3 shows the results of EDS spectroscopy analysis, which lists the results of chemical composition measurement of each phase in Figure 9. The results show that measuring points A and G are mainly composed of Ag elements. Point H is mainly composed of a large number of Ti elements and a small number of other elements, which is considered to be a Ti-based solid solution containing some other elements, hereinafter referred to as Ti(ss). Similarly, the structure of point B, C, D, E consists of a large amount of Ti and a small amount of Cu elements and a small number of other elements. The increase of Cu content indicates that Ti_2_Cu is produced by the interaction between AgCuTi filler and substrate at the interface, which has been confirmed by later interfacial XRD (D8 Advance, Bruker, Germany; the microfocus X-ray(IµS) source) results. Points F, J and I are identified as the matrix TC4, but as shown in Figure 10, the prior wetting structure of the matrix is mainly composed of needle-like α′ martensite structure, which transforms into rod-like after wetting α phase. This can be attributed to that when the temperature rises to the two-phase transforming temperature during wetting, α′ phase transforms into β. However, as the temperature of 900 °C is lower than that of β phase transition lines, α phase does not completely transform into β phase. 

**Figure 11:**
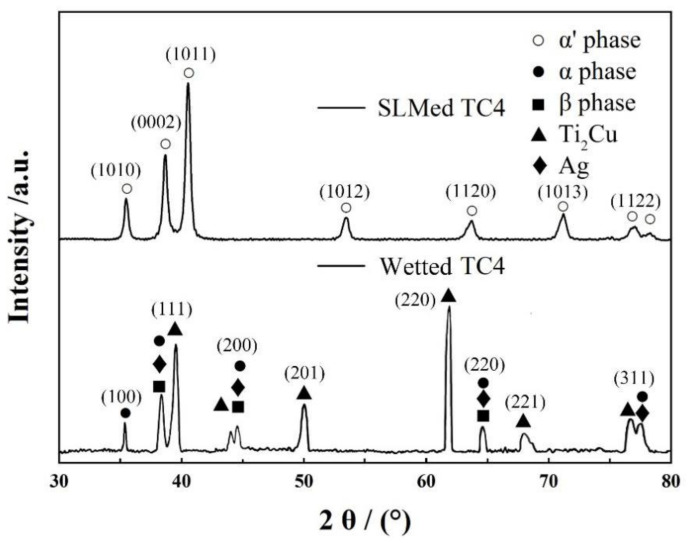
X-ray diffraction (XRD) patterns of wetting interface and matrix.

The wetted and solidified SLMed TC4 is cut along the central line, the cross-section area is selected across the interface, and the diffraction pattern shown in Figure 11 is obtained by XRD. Figure 11 shows the XRD pattern of SLMed TC4 after wetting. It can be seen that there are only some defects in the XRD pattern of TC4 matrix formed by SLM α’. The peak of martensite is similar to that of Krakhmalev et al. [33]. However, after being wetted by AgCuTi filler metal, the diffraction peaks of Ag and Ti_2_Cu appear, which are different from the original ones α′ phase change to α and a small amount to β, which also confirms the above organizational analysis.

### 3.3. Comparison with AgCuTi/Rolled TC4 System

**Figure 12:**
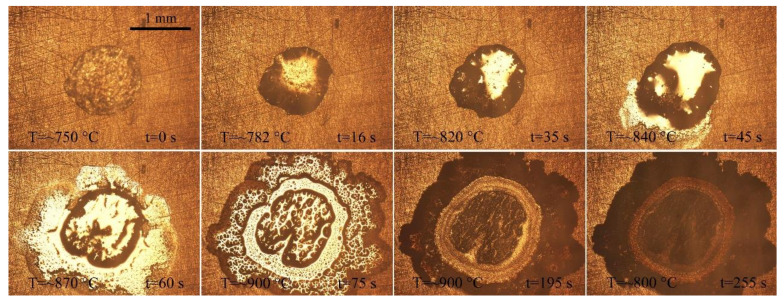
Typical instantaneous photos of AgCuTi wetting and spreading process on rolled TC4 substrate at 900 °C.

**Figure 13:**
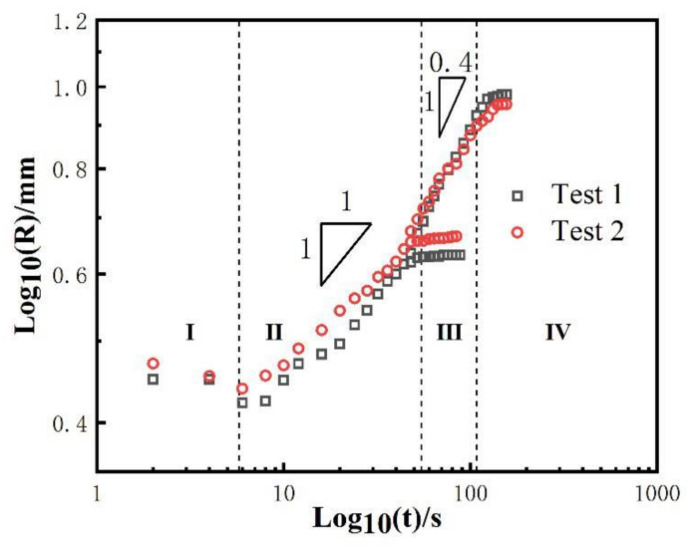
Logarithmic relationship between equivalent spreading radius and time of AgCuTi/rolled TC4 wetting system at 900 °C.

For comparison, the AgCuTi filler metal was used for wetting on the traditionally rolled TC4 substrate. According to the results in Section 3.1.1, the typical conditions (900 °C, 120 s) with good wetting effect were selected. The peak temperature was selected as 900 °C and the holding time was 120 s. Figure 12 shows a series of representative instantaneous images during spreading. This is similar to the wetting morphology of the AgCuTi/SLMed TC4 system. In addition, the logarithmic relationship between the equivalent spreading radius and time of the AgCuTi filler metal during wetting is shown in Figure 13. By observing the experimental video, it can be seen that wetting process of AgCuTi on the rolled TC4 system at 900 °C is similar to that of the AgCuTi on the SLMed TC4. Similarly, the wetting spreading process on the traditionally rolled TC4 can also be divided into four stages: (i) initial spreading; (ii) rapid spreading; (iii) precursor film spreading; (iv) the stage of approaching balance. The rapid spreading stage of AgCuTi filler on the traditionally rolled TC4 substrate also can be conformed to the R^n^ ~ t model, where n = 1, and n = 0.4 for the spreading of precursor film, so the intrinsic processes are similar. Although the phases of the base metal of the two systems are different before wetting (The SLMed TC4 is mainly composed of α′ while the rolled TC4 mainly consists of α + β), the element compositions of the two matrixes (both are Ti-6Al-4V) are similar after high-temperature wetting, which leads to the same chemical reactions and similar wetting kinetics in the process of wetting and spreading. 

## 4. Conclusions

In this study, the wetting and spreading process was observed and recorded in situ in the protective atmosphere of ultra-high-purity argon (99.999%) assisted by a high-temperature hot stage microscope. The effects of temperature and holding time on the wetting spreading kinetics and interfacial structure of the AgCuTi/SLMed TC4 system were studied The wetting and spreading properties of the AgCuTi/rolled TC4 system were compared as a reference. 

Based on the analysis of the experimental results, the conclusions are as follows:

1. Temperature has a great influence on the AgCuTi/SLMed TC4 system. With the increase of brazing temperature, the final wetting area of AgCuTi filler on SLMed TC4 gradually increases. Precursor film appears at the front edge of the triple line at temperatures of 850 °C and 900 °C. As the temperature increases, the spreading of the precursor film becomes faster, especially at 950 °C.
2. The wetting process of AgCuTi filler metal on SLMed TC4 is similar at 850 °C and 900 °C and can be divided into four stages: (i) initial spreading stage; (ii) rapid spreading stage; (iii) the precursor film spreading stage; (iv) the stage of approaching balance. However, at 950 °C, the increase of temperature promotes the wetting of AgCuTi on SLMed TC4 substrate, which can also be divided into four stages: (i) initial spreading stage; (ii) rapid spreading stage; (iii) slow spreading and the precursor film spreading stage, when two processes take place at the same time; (iv) the stage of approaching balance.
3. Holding time slightly promotes the spreading process. Liquid filler metal spreads rapidly during the heating stage and slowly during the dwell process. The prolongation of holding time promotes the slow spreading stage.
4. The kinetics of wetting of the AgCuTi/SLMed TC4 system can be analyzed by R^n^ ~ t model. The n value of the rapid spreading stage is 1, which belongs to the reaction-limited wetting. The n values of the precursor film spreading stage are 0.44, 0.4 and 0.35, respectively. Temperature plays an important role in the precursor film spreading process.
5. The wetting spreading of AgCuTi/rolled TC4 system is similar to that of the AgCuTi/SLMed TC4 system in terms of phenomena and wetting kinetics.

## Figures and Tables

**Table 1:** Selective laser-melting (SLM) parameters.

Process Parameters	Values
Laser energy density (*VED*)	69.5 J·mm^−3^
Laser power (*P*)	275 W
Laser scanning speed (*v*)	1100 mm·s^−1^
Printing layer thickness (*h*)	0.03 mm
Overlap spacing (*l*)	0.12 mm

**Table 2:** Chemical composition of Ti-6Al-4V alloy.

wt.%							
**Al**	**V**	**Fe**	**C**	**N**	**O**	**H**	Ti
5.5~6.8	3.5~4.5	≤0.30	≤0.10	≤0.05	≤0.20	≤0.015	Margin

**Table 3:** Results of EDS analysis.

Points	Main Elements (at.%)					Possible Phase
	Ag	Cu	Ti	Al	V	
A	97.66	2.34	-	-	-	Ag(ss)
B	8.37	14.91	71.02	5.7	-	Ti_2_Cu + Ti(ss)
C	3.42	7.56	75.02	9.23	4.68	Ti_2_Cu + Ti(ss)
D	2.48	8.89	74.82	10.08	3.73	Ti_2_Cu + Ti(ss)
E	9.46	18.28	66.54	4.39	1.33	Ti_2_Cu + Ti(ss)
F	-	-	85.96	11.21	2.83	α-Ti
G	95.98	0.51	3.51	-	-	Ag(ss)
H	4.56	2.11	82.34	10.99	-	Ti(ss)
I	1.25	2.63	80.19	13.02	3.01	α-Ti
J	-	1.99	82.57	12.65	2.79	β-Ti

## Data Availability

No new data were created or analyzed in this study. Data sharing is not applicable to this article.

## References

[1] Huang J. (2018). Study of Microstructure and Properties of TC4 Alloy by Selective Laser Melting. Master’s Thesis.

[2] Su Y., Wu B., Wang X. (2016). Research on Further Application of Additive Manufacturing Technology on Aviation Equipment. Aeronaut. Manuf. Technol..

[3] Yan X., Ruan X. (2016). Application and development of additive manufacturing technology in aeroengine. Aeronaut. Manuf. Technol..

[4] Gurrappa I. (2003). Characterization of titanium alloy Ti-6A1-4V for chemical, marine and industrial applications. Mater. Charact..

[5] Vastola G., Zhang G., Pei Q.-X., Zhang Y. (2016). Modeling the Microstructure Evolution during Additive Manufacturing of Ti6Al4V: A Comparison between Electron Beam Melting and Selective Laser Melting. JOM.

[6] Do D.-K., Li P. (2016). The effect of laser energy input on the microstructure, physical and mechanical properties of Ti-6Al-4V alloys by selective laser melting. Virtual Phys. Prototyp..

[7] Xia C., Zhao M., Sun W., Li H., Liu P. (2019). Microstructure and Properties of 3D Printed Inconel 718 Joint Brazed with BNi-2 Amorphous Filler Metal. Mater. Res..

[8] Prashanth K., Damodaram R., Scudino S., Wang Z., Rao K.P., Eckert J. (2014). Friction welding of Al-12Si parts produced by selective laser melting. Mater. Des..

[9] Prashanth K., Damodaram R., Maity T., Wang P., Eckert J. (2017). Friction welding of selective laser melted Ti6Al4V parts. Mater. Sci. Eng. A.

[10] Nahmany M., Rosenthal I., Benishti I., Frage N., Stern A. (2015). Electron beam welding of AlSi10Mg workpieces produced by selected laser melting additive manufacturing technology. Addit. Manuf..

[11] Tillmann W., Henning T., Wojarski L. (2018). Vacuum brazing of 316L stainless steel based on additively manufactured and conventional material grades. IOP conference series. Mater. Sci. Eng..

[12] Yu H., Li F., Yang J., Shao J., Wang Z., Zeng X. (2018). Investigation on laser welding of selective laser melted Ti-6Al-4V parts: Weldability, microstructure and mechanical properties. Mater. Sci. Eng. A.

[13] Akselsen O.-M. (1992). Advances in brazing of ceramics. J. Mater. Sci..

[14] Saiz E., Cannon R.-M., Tomsia A.-P. (2000). Reactive spreading: Adsorption, ridging and compound formation. Acta Mater..

[15] Saiz E., Tomsia A.-P., Suganuma K. (2003). Wetting and strength issues at Al/α-alumina interfaces. J. Eur. Ceram. Soc..

[16] Villanueva W., Boettinger W., McFadden G., Warren J. (2012). A diffuse-interface model of reactive wetting with intermetallic formation. Acta Mater..

[17] Yin L., Murray B.-T., Singler T.-J. (2006). Dissolutive wetting in the Bi-Sn system. Acta Mater..

[18] Yu X., Yang J., Yan M., Hu X.W., Li Y.L. (2016). Kinetics of wetting and spreading of AgCu filler metal over Ti–6Al–4V substrates. J. Mater. Sci..

[19] Liu J., Liu G.-P., Ouyang H., Li Y.-L., Yan M., Pecht M. (2020). Wetting Kinetics and Microstructure Analysis of BNi2 Filler Metal over Selective Laser Melted Ti-6Al-4V Substrate. Materials.

[20] Huang X., Lei M., Li X., Li Y. (2019). Effects of surface roughness on the TiAl/AgCu/TiAl brazing quality. Mater. Sci. Technol..

[21] Li Y., Liu W., Sekulic D.-P., He P. (2012). Reactive wetting of AgCuTi filler metal on the TiAl-based alloy substrate. Appl. Surf. Sci..

[22] Liu G., Li Y., Long W., Hu X., Cao J., Yan M. (2019). Wetting kinetics and spreading phenomena of the precursor film and bulk liquid in the AgCuTi/TC4 system. J. Alloy. Compd..

[23] Zhao H., Wang H.Q., Sekulic D.P., Qian Y.Y. (2009). Spreading Kinetics of Liquid Solders over an Intermetallic Solid Surface. Part 1: Eutectic Lead Solder. J. Electron. Mater..

[24] Kozlova O., Voytovych R., Protsenko P., Eustathopoulos N. (2010). Non-reactive versus dissolutive wetting of Ag–Cu alloys on Cu substrates. J. Mater. Sci..

[25] Tillmann W., Pfeiffer J., Sievers N., Boettcher K. (2015). Analyses of the spreading kinetics of AgCuTi melts on silicon carbide below 900°C, using a large-chamber SEM. Colloids Surf. A Physicochem. Eng. Asp..

[26] Landry K., Kalogeropoulou S., Eustathopoulos N. (1998). Wettability of carbon by aluminum and aluminum alloys. Mater. Sci. Eng. A.

[27] Mortensen A., Drevet B., Eustathopoulos N. (1997). Kinetics of diffusion-limited spreading of sessile drops in reactive wetting. Scr. Mater..

[28] Landry K., Eustathopoulos N. (1996). Dynamics of wetting in reactive metal/ceramic systems: Linear spreading. Acta Mater..

[29] Li Y., Wang Z., Li X., Lei M. (2020). Effect of temperature and substrate surface roughness on wetting behavior and interfacial structure between Sn–35Bi–1Ag solder and Cu substrate. J. Mater. Sci. Mater. Electron..

[30] Xian A.-P. (1993). Precursor film of tin-based active solder wetting on ceramics. J. Mater. Sci..

[31] Qla B., Feng Q.-B., Ran S.-C. (2014). Characteristics of precursor film in the wetting of Zr-based alloys on ZrC substrate at 1253K. Thin Solid Films.

[32] Lin Q., Jin P., Cao R., Chen J. (2016). Reactive wetting of low carbon steel by Al 4043 and 6061 alloys at 600–750 °C. Surf. Coat. Technol..

[33] Krakhmalev P., Fredriksson G., Yadroitsava I., Kazantseva N., du Plessis A., Yadroitsev I. (2016). Deformation Behavior and Microstructure of Ti6Al4V Manufactured by SLM. Phys. Procedia.

